# Macrophage, a potential targeted therapeutic immune cell for cardiomyopathy

**DOI:** 10.3389/fcell.2022.908790

**Published:** 2022-09-30

**Authors:** Ganyi Chen, Hongwei Jiang, Yiwei Yao, Zhonghao Tao, Wen Chen, Fuhua Huang, Xin Chen

**Affiliations:** ^1^ Department of Thoracic and Cardiovascular Surgery, Nanjing First Hospital, Nanjing Medical University, Nanjing, Jiangsu, China; ^2^ Shanghai Pulmonary Hospital, Tongji University, Shanghai, China

**Keywords:** macrophage, heart failure, cardiomyopathy, phenotype, resident macrophages

## Abstract

Cardiomyopathy is a major cause of heart failure, leading to systolic and diastolic dysfunction and promoting adverse cardiac remodeling. Macrophages, as key immune cells of the heart, play a crucial role in inflammation and fibrosis. Moreover, exogenous and cardiac resident macrophages are functionally and phenotypically different during cardiac injury. Although experimental evidence has shown that macrophage-targeted therapy is promising in cardiomyopathy, clinical translation remains challenging. In this article, the molecular mechanism of macrophages in cardiomyopathy has been discussed in detail based on existing literature. The issues and considerations of clinical treatment strategies for myocardial fibrosis has also been analyzed.

## Introduction

Although great progress has been made in preventing and treating cardiovascular diseases, it still has the highest morbidity and mortality worldwide (Roth et al., 2020). The development of cardiovascular disease to the end-stage leads to heart failure, one of the main causes of cardiovascular death (Crespo-Leiro et al., 2018; Roth et al., 2020). Cardiomyopathy is the major cause of heart failure. Cardiomyopathy is defined as a heterogeneous myocardial disease with inappropriate hypertrophy or dilation of the ventricle (Brieler et al., 2017). Besides, the definition and classification of cardiomyopathy has considerably changed, including primary and secondary categories (McKenna et al., 2017). The categories are subdivided into varied phenotypes including dilated, hypertrophic, and restrictive patterns. Heart transplantation is the only effective treatment for heart failure (Truby and Rogers, 2020). Therefore, it is important to understand the pathological development of cardiomyopathy.

The heart comprises various cells, including cardiomyocytes, smooth muscle cells, fibroblasts, endothelial cells, and various immune cells (Frangogiannis, 2019; Peet et al., 2020). Monocytes are a key component of immune cells. They can produce various inflammatory cytokines such as IL-1, TNFα and IL-6, by exposing endogenous damage-associated molecular patterns (DAMPs) after cell injury. Chemokines can recruit monocytes from the blood and spleen to the heart and activate their separation into macrophages (Honold and Nahrendorf, 2018). Macrophages have in recent years become a key research area in cardiovascular diseases. Macrophages are central regulators of the immune system. They can activate and proliferate lymphocytes to generate innate and adaptive immune responses for defense, inflammation, and tissue recovery (Varol et al., 2015; Wynn and Vannella, 2016; Shapouri-Moghaddam et al., 2018).

Macrophages perform different functions during myocardial damage. In the myocardial infarction/ischemia reperfusion injury, pro-inflammatory macrophages play a dominant role in the early stages, repair macrophages play an important role as the disease progresses. In other cardiomyopathy, macrophages exhibit different functional phenotypes under different stimuli. Macrophages, as primary immune cells, can regulate repair and healing after myocardial injury. Therefore, they are potential target immune cell for heart failure prevention and therapeutic modalities (Sager et al., 2016; Dick et al., 2019; Zhao et al., 2019). In this paper, we focused on the main function of macrophages in cardiomyopathy.

## Functions and phenotype of macrophages

Macrophages are key elements of innate and adaptive immune responses. Macrophages regulate inflammation and homeostasis during organ development (Wynn et al., 2013). For mant years, quite a few people were sure that all human macrophages were differentiated from blood monocytes after penetrating blood vessels (van Furth and Cohn, 1968), however, Epelman et al. refute this hypothesis (Epelman et al., 2014a). Currently, the yolk sac, fetal liver and bone marrow hematopoietic cells are the main sources of tissue-macrophages (Hogg et al., 2020). In fact, each organ has the unique macrophages combination. In addition, monocyte-derived macrophages release proinflammatory factors to increase the inflammatory response and tissue-macrophages assist in host defense and tissue remodeling. Macrophages have a highly plastic phenotype dependent on the activation (*in vitro*) or the microenvironment (*in vivo*) under appropriate environmental stimuli (Zhang et al., 2021). Notably, macrophages act as a pro-inflammatory or an anti-inflammatory subtype to protect the body from injury.

**FIGURE 1:**
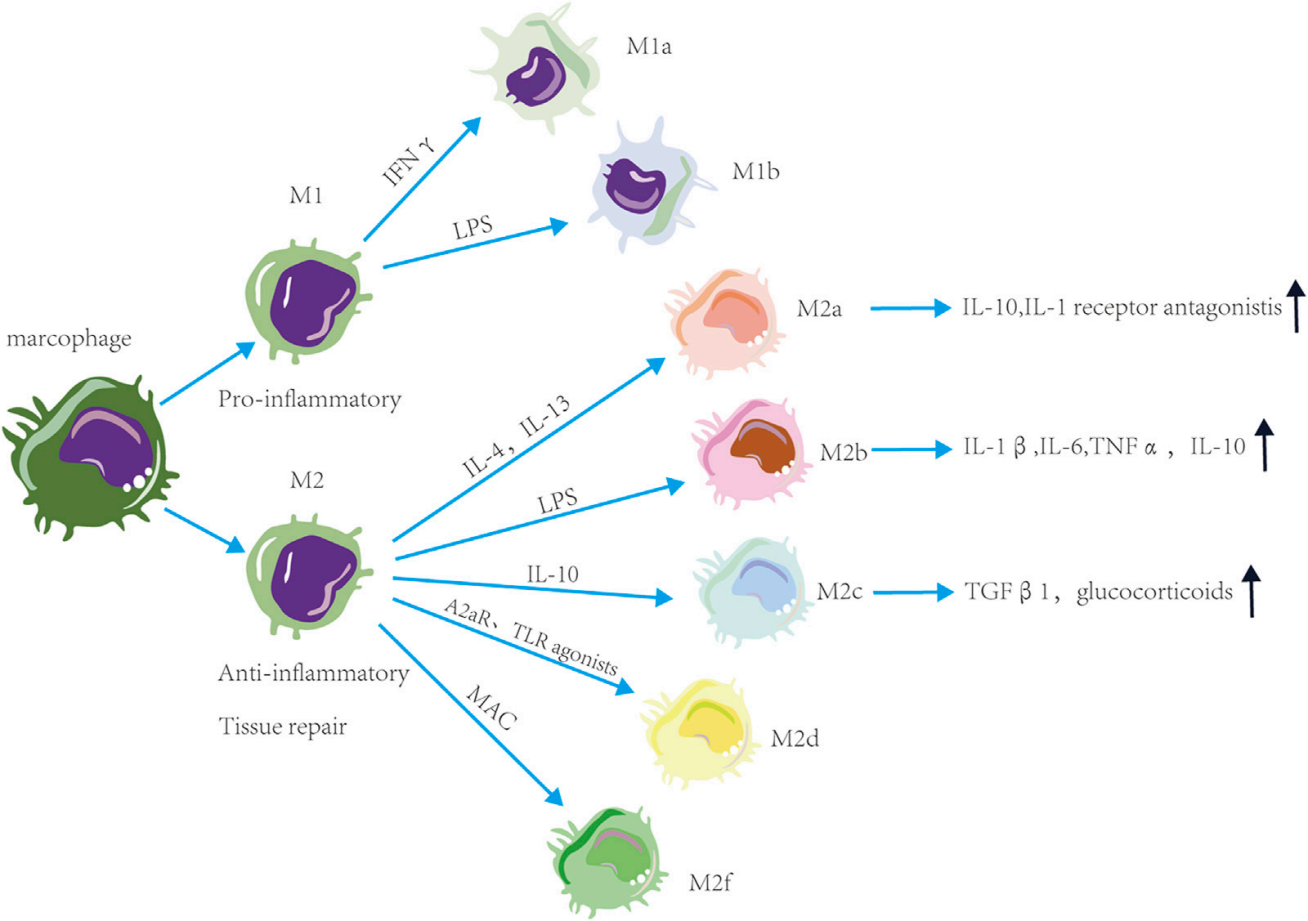
Functions and phenotype of macrophages. Macrophages are classified into M1 and M2 based on the stimulation type and function. M1 macrophages have pro-inflammatory activity, M2 macrophages have anti-inflammatory and tissue repair activity. These macrophages are divided into subtypes based on the stimulation. Proinflammatory M1 macrophages are divided into M1a macrophages stimulated by IFNγ and M1b macrophages stimulated by LPS. Anti-inflammatory M1 macrophages are divided into M2a macrophages stimulated by IL-4 and IL-13, M2b macrophages stimulated by LPS, M2c macrophages stimulated by IL-10, M2d macrophages stimulated by A_2A_R and TLR agonists and M2f macrophages stimulated by macrophage apoptotic clearance. M2a macrophages can suppress the inflammatory responses. M2b macrophages have pro- and anti-inflammatory activity. M2c macrophages can secrete TGFβ1 and glucocorticoids.

Macrophage exposure to excessive stimuli may make macrophages have different functions and cell surface markers. Macrophages are classified into M1 and M2 based on the stimulation type, surface molecule expression, function, and secretion profile (Figure 1). Proinflammatory M1 macrophages have anti-pathogen activity. M1a macrophages are stimulated by IFNγ, while M1b macrophages are stimulated by LPS (Mukhopadhyay et al., 2004; Martinez et al., 2008). Anti-inflammatory M2 macrophages promote tissue repair. M2a macrophages with a predominantly anti-inflammatory phenotype are polarized by IL-4 and IL-13 and secrete IL-10 and IL-1 receptor antagonists to suppress the inflammatory responses (Lee et al., 2001; Sierra-Filardi et al., 2010; Jetten et al., 2014). The M2b macrophages with pro- and anti-inflammatory phenotypes produce IL-1β, IL-6, TNFα, and IL-10 vi LPS stimulation (Mosser, 2003). M2c macrophages activated by IL-10 can secrete transforming growth factor 1 (TGFβ1) and glucocorticoids (Mosser and Edwards, 2008). M2d macrophages are activated by adenosine A_2A_ receptor (A_2A_R) agonists and toll-like receptor (TLR) agonists (Ferrante et al., 2013). M2f cells macrophages are stimulated by macrophage apoptotic clearance (MAC) (O'Rourke et al., 2019). In summary, the polarization of macrophages is closely related to changes in glycolysis (M1-like) and oxidative phosphorylation (M2-like).

## Origin of heart resident macrophages

Although the origin of tissue macrophages has been controversial over the past years, tissue macrophages are currently a hot research area. Resident tissue macrophages in most adult organs develop from fetal monocytes and can be locally sustained throughout the life cycle (Sawai et al., 2016; Bajpai et al., 2018). Resident cardiac macrophages have an essential role in homeostasis, cardiac function, and tissue repair. The four populations in normal mouse hearts have varying levels of Ly6C, CD11c, CCR2, and the major histocompatibility complex (MHCII) (Epelman et al., 2014a). Single-cell RNA sequencing of cardiac macrophages used an unbiased approach to confirm the presence of three subpopulations. Cardiac macrophages were of M2 type during the stabilization period, accompanied by high expression of Ly6C, and had a repair function. Interestingly, cardiac macrophages for CCR2 are located in the myocardial wall. They are essential for the reconstruction of the original coronary by secreting pro-angiogenic signals. CCR2-macrophages develop in the heart during the embryonic. They are maintained under homeostatic conditions independently of peripheral blood mononuclear cell infiltration, and promote electrical conduction within the atrioventricular node function (Bajpai et al., 2019).

## Role of macrophages on myocarditis

Myocarditis is an inflammatory process affecting the muscle tissue (myocardium) of the heart (Cooper, 2009). Autopsy studies in the general population have shown that myocarditis is the main cause of sudden death and an early cause of cardiomyopathy (Ammirati et al., 2020). Viral infection is a major cause of myocarditis. It also activates the immune system to cause myocardial inflammation, necrosis, the release of DAMPs, and ventricular dysfunction by directly killing virus-infected cardiomyocytes and increasing the production of inflammatory cytokines. As a result, it affects the function of cardiomyocytes, eventually leading to the occurrence of dilated cardiomyopathy (Luo et al., 2010; Pollack et al., 2015). The incidence of viral myocarditis is closely associated with various viral infections, such as enteroviruses, influenza viruses, and cytomegalovirus. The pathogenesis of viral myocarditis has three stages: acute viremia stage, subacute infiltration stage, and chronic stage. Heart transplantation is the main treatment method for myocarditis clinically.

Monocytes and macrophages are the major constituents of inflammatory cell infiltration in human myocarditis and may play an immunomodulatory role. Cardiac injury caused by myocarditis leads to early Ly6Chi inflammatory macrophage recruitment (Barin et al., 2012). Macrophages affect the cardiac immune microenvironment by secreting proinflammatory cytokines, including TNF-α, IL-1β, IL-6, and chemokine CCL2. Macrophages can also modulate the strength, bias, and persistence of subsequent adaptive immune responses. B cells can aggravate myocardial injury by secreting cytokines and promoting monocyte recruitment and CCR2 macrophage infiltration (Bajpai et al., 2019; Heinrichs et al., 2021). M2 macrophages inhibit inflammatory responses and promote cardiac fibrotic healing. Therefore, it is a hallmark of the transition from acute myocarditis to chronic pathological remodeling, with macrophage replacement by profibrotic myofibroblasts.

Coronavirus is a class of enveloped, positive, single-stranded, and highly diverse RNA viruses. SARS coronavirus 2 that causes COVID-19 is a novel β-coronavirus with high sequence homology to the early SARS coronavirus that caused the SARS outbreak (Zhou et al., 2021). The new coronavirus causes many and very serious sequelae, especially in the cardiovascular aspect. This indicates that troponin-I, a biomarker of myocardial damage, is elevated after myocardial injury in COVID-19 patients (Nishiga et al., 2020; Zaninotto et al., 2020). Moreover, COVID-19 patients have other cardiovascular complications, such as acute myocardial infarction, myocarditis, and heart failure. Myocarditis develops longer (up to 10–15 days) after the onset of symptoms in COVID-19 patients. A 32 adaptive T-cell-mediated immunity may play a key role in the development of myocardial inflammation after 2 weeks of COVID-19 onset. Notably, an increase in the proinflammatory CCR6+ and Th17 in CD4^+^ T cells, the main inflammatory mediator of myocarditis, has been reported in severe cases (Golovkin et al., 2021).

SARS-CoV2 can replicate in cardiomyocytes derived by human-induced pluripotent stem cell (hiPSC) and induce cytotoxic effects to eliminating cardiomyocyte beating (Sharma et al., 2020; Lee et al., 2021). Another study showed that SARS-CoV2 utilizes the ACE2 receptors for internalization *via* the transmembrane protease serine 2 protease (Hatmal et al., 2020). Virus-induced downregulation of ACE2 may attenuate the function, reduce anti-inflammatory effect, and elevate the effect of AngII in susceptible patients (Perez-Bermejo et al., 2020). Yang et al. have found macrophages mediates myocardial injury caused by SARS-CoV2. Macrophages can increase TNFα and IL-6 production and active JAK/STAT pathway to exacerbate oxidative stress (Yang et al., 2021). Meanwhie, Husam Jum’ah et al. have demonstrated the density of macrophages is positively correlated with myocardial apoptosis (Jum’ah et al., 2022). However, the infectivity of cardiac resident macrophages is unknown.

## The role of macrophages on cardiomyopathy

Current researches have demonstrated that immune cells play a crucial role in the formation and prognosis of cardiomyopathy. Cardiomyopathy is segmented into primary cardiomyopathy and secondary cardiomyopathy (Burke et al., 2016; Brieler et al., 2017). Primary cardiomyopathy includes dilated cardiomyopathy, hypertrophic cardiomyopathy, restrictive cardiomyopathy, and indeterminate cardiomyopathy while secondary cardiomyopathy involves cardiomyopathy caused by systemic disease. This article focuses on the role of macrophages in three types of cardiomyopathy: ischemic cardiomyopathy, dilated cardiomyopathy, and diabetic cardiomyopathy.

## Ischemic cardiomyopathy

Ischemic cardiomyopathy (ICM), a special type of an advanced stage of coronary heart disease, is a long-term myocardial ischemia caused by coronary atherosclerosis. It results in diffuse myocardial fibrosis and a disease-like clinical syndrome. With the increasing incidence of coronary heart disease, the harm caused by ICM to human health is becoming more and more serious. Myocardial infarction and myocardial defect-reperfusion injury are major causes of ICM. Ischemic damage occurs in the areas oxygenated by blocked arteries in the heart. Thrombolysis and percutaneous coronary intervention are usually used clinically to restore coronary blood flow. However, ischemia/reperfusion injury is still a common complication (Fan et al., 2019). This article focuses on the interaction between IR and macrophages.

Peripheral monocyte mobilization and infiltration in cardiovascular disease is primarily considered a maladaptive response associated with adverse outcomes including infarct dilation, left ventricular systolic dysfunction, and formation of atherosclerotic plaques (Bajpai et al., 2019). Several evidences have shown that the immune response has a central role in IR injury, characterized by the recruitment and activation of the innate and adaptive immune cells (Fan et al., 2019; Wei et al., 2019). For example, peripheral blood CD14^+^ monocyte abundance is associated with larger infarct size and worsening of left ventricular systolic function in myocardial infarction (Kaczorowski et al., 2009).

CCR2+ macrophages increase MyD88-dependent CCL2 production in mice, thus promoting monocyte recruitment. In contrast, CCR2-resident macrophages impair monocyte recruitment (Li et al., 2016). Expression profiling has shown that CCR2+ macrophages are required for neutrophil extravasation into the myocardium (Li et al., 2016). Furthermore, studies have shown that the expression of type I interferon-stimulated genes in resident CCR2-macrophages after myocardial injury is significantly different compared with recruited CCR2+ macrophages, suggesting that CCR2-resident macrophages are responsible for the production of myocardial infarction during myocardial infarction (Calcagno et al., 2020). The Ly6Chi population associated with the M1 pro-inflammatory macrophage phenotype is recruited through increased CCL2/CCR2 chemokine/monocyte receptor interaction and endothelial adhesion molecule expression in the early stage of MI inflammation (Duncan et al., 2020). The late Ly6Clow population associated with the M2 ″healing” phenotype infiltrated the myocardium between days 4 and 7 post-MI(54). These Ly6Clow macrophages are reparative and non-inflammatory and can promote myofibroblast accumulation, angiogenesis, and collagen deposition. Inhibition of monocyte recruitment by blocking the CCL2 and CCR2 signaling pathways reduces excessive inflammation, myocardial infarction and atherosclerosis is protective in a sclerosis-like mouse model (Tsou et al., 2007).

## Dilated cardiomyopathy

Dilated cardiomyopathy is a primary heart muscle disease with an unknown cause. The disease is characterized by left or right ventricle or bilateral ventricular enlargement and ventricular systolic hypofunction, with or without congestive heart failure (Japp et al., 2016). The disease is caused by various factors, including genetic factors, viral infections, and autoimmune phenomena. Heart transplantation is the only treatment for the disease. However, it is also difficult to find the heart. The adverse reactions, such as postoperative anti-rejection reactions and decreased renal function, also affect patient survival. Therefore, understanding the pathophysiological process of dilated cardiomyopathy is necessary to explore new treatment methods and control its occurrence and development in the early stage. This article focused on the relationship between doxorubicin-mediated dilated cardiomyopathy and macrophages.

Doxorubicin-induced cardiomyopathy (DIC) is characterized by decreased contractility, rhythmic relaxation, and adverse cardiac remodeling (Chatterjee et al., 2010). Cardiomyocyte apoptosis, fibrosis, hypertrophy, and cell vacuolization are the most classic physiological and pathological changes of cardiomyopathy caused by adriamycin (Lipshultz et al., 1991; Zhao et al., 2018). Doxorubicin causes cardiomyocyte death through DNA damage and production of reactive oxygen species (ROS), which trigger mitochondrial dysfunction (Enomoto et al., 2021). Cardiomyocytes contain several mitochondria, thus providing metabolic energy and mechanical demands for the heart to eliminate dysfunctional mitochondria, thus maintaining a healthy state of cardiomyocytes (Garbern and Lee, 2021). Depletion of cardiac macrophages affects mitochondrial elimination, accumulation of abnormal mitochondria in myocardial tissue, activation of inflammasomes, metabolism, and ventricular dysfunction (Nicolas-Avila et al., 2020).

Macrophages in non-ischemic cardiomyopathy respond to injury-related molecular patterns and chemokine signaling, then differentiate into defined phenotypes guided by local cytokine signaling. Resident cardiac macrophages are associated with adaptive cardiac response to stress overload (Epelman et al., 2014b). Recent studies have suggested that DIC may be involved in M1 macrophage infiltration, induction of inflammation, and pathogenesis. For instance, Qi Chen suggested that peripheral pro-inflammatory monocyte-derived cardiac macrophages predominate during DIC. However, cardiac macrophages eventually mobilize and self-renew in response to doxorubicin and reduce adverse cardiac remodeling (Zhang et al., 2020).

Although a good beginning has been made in the study of macrophages in the DIC, there are still some important problems to be solved. This includes how to adjust the immune strategy to respond to the activation of recruited macrophages and the survival of cardiac resident macrophages.

## Diabetic cardiomyopathy

Diabetic cardiomyopathy occurs in diabetic patients. It leads to extensive focal necrosis of the myocardium, eventually causing heart failure, arrhythmia, and cardiogenic shock (Dillmann, 2019). Its pathogenesis is mainly manifested as chronic hyperglycemia, insulin resistance, and chronic inflammation. Higher white blood cell counts can predict the risk of cardiovascular disease in diabetic patients, suggesting that these cells play a key role in the worsening of diabetes-related cardiovascular disease (Zhang et al., 2019).

Diabetic patients have increased immune cell infiltration and monocyte activity (Morano et al., 2007; Wu et al., 2020). The heart is primarily dependent on glycolysis during decompensated heart failure. Its central failure accounts for the activation of HIF-1α, which induces glycolysis and transcription of pro-inflammatory genes (Holscher et al., 2012). Macrophages can lead to protein glycation and advanced glycation end products due to excess glucose levels, activating the NF-κB pathway and the production of inflammatory cytokines, leading to microvascular and macrovascular complications (Volz et al., 2010; Bezold et al., 2019). Moreover, blood glucose level elevation increases superoxide production, which may directly lead to cell damage (Iannantuoni et al., 2020).

## The function of macrophages in cardiac remodeling and fibrosis

Myocardial injury causes myocardial fibrosis. Myocardial injury makes fibroblasts overactive, and thus their secreted extracellular matrix is excessively deposited in the cardiac interstitium and around blood vessels. As a result, it leads to myocardial wall thickening, contraction, relaxation and dysfunction, thus reducing cardiac function (Fan et al., 2012). Fibroblasts are key in the homeostasis of the extracellular matrix under physiological conditions. The extracellular matrix provides a structural scaffold for cardiomyocytes. The extracellular matrix also wraps the cardiomyocytes and stabilizes the mechanical force and electrical conduction between the cardiomyocytes. Way to ensure the structural and functional integrity of the heart (Camelliti et al., 2005; Porter and Turner, 2009; Souders et al., 2009). Pathophysiological stimuli, such as inflammation and pressure overload, can also cause myocardial injury. For instance, the massive death of myocardial cells triggers a strong inflammatory response. As a result, fibroblasts are rapidly activated and transformed into fibroblasts, which secrete several extracellular cells (Ivey et al., 2018). Matrix protein forms a collagen-based scar to replace cardiomyocytes to prevent rupture of the heart, thus protecting heart function. However, prolonged or excessive fibrosis can lead to reduced cardiac compliance, left ventricular systolic and diastolic dysfunction, and eventually congestive heart failure.

Macrophages play a key role in cardiac remodeling. Myocardial injury recruits several subsets of peripheral blood monocytes/macrophages differentiated from the bone marrow or spleen, including pro- and anti-inflammatory cells. These cells secrete pro- or anti-inflammatory factors, pro-angiogenic factors, pro-fibrotic repair factors, and promote the repair response of the heart by phagocytosing and removing necrotic or apoptotic cardiomyocytes (Aurora et al., 2014; Lavine et al., 2014; Hulsmans et al., 2018). The degree of inflammation is significantly associated with the severity of heart failure. Pro-inflammatory macrophages mainly secrete a large number of pro-inflammatory cytokines and chemokines such as IL-6, IL-1β, in the early stage of myocardial infarction. As a result, anti-inflammatory/reparative macrophages dominate by secreting high levels of anti-inflammatory and angiogenic factors, such as vascular endothelial growth factor (VEGF), fibroblast growth factor 2 (FGF2), and transforming growth factor-beta (TGF-β), promoting cardiac repair and maintain cardiac structural integrity by inducing myofibroblast activation, collagen deposition, and development of new blood vessels (Nahrendorf et al., 2007; Gombozhapova et al., 2017; Mouton et al., 2018). Recent studies have shown that macrophages can suppress excessive cardiac fibrosis by secreting oncostatin-m (OSM) through hypoxic signaling (Abe et al., 2019). Activation of the YAP/TAZ pathway enhances macrophage-mediated fibrosis and tissue remodeling in cardiomyocytes (Mia et al., 2020). These evidences further demonstrate the role of macrophage-mediated fibrosis after myocardial injury.

Besides recruited macrophages, resident macrophages in the heart also play a key role in the fibrosis process after myocardial injury. Studies have shown that cardiac resident CCR2-macrophages can promote cardiac recovery by promoting the proliferation of cardiomyocytes and angiogenesis (Lavine et al., 2014). However, they are replaced by recruited pro-inflammatory macrophages after myocardial injury and lack repair activity. Recent studies have demonstrated that cardiac resident CCR2-macrophages stimulate angiogenesis while inhibiting fibrosis during cardiac pressure overload (Revelo et al., 2021). Similarly, miRNA21 in macrophages can promote fibroblast activation in a heart failure model *via* paracrine, leading to myocardial fibrosis (Verma et al., 2017). However, the specific molecular mechanism of the inhibitory effect of cardiac resident CCR2-macrophages on fibrosis requires further research.

## Potential therapeutic strategy of macrophages for myocarditis and cardiomyopathy

**FIGURE 2:**
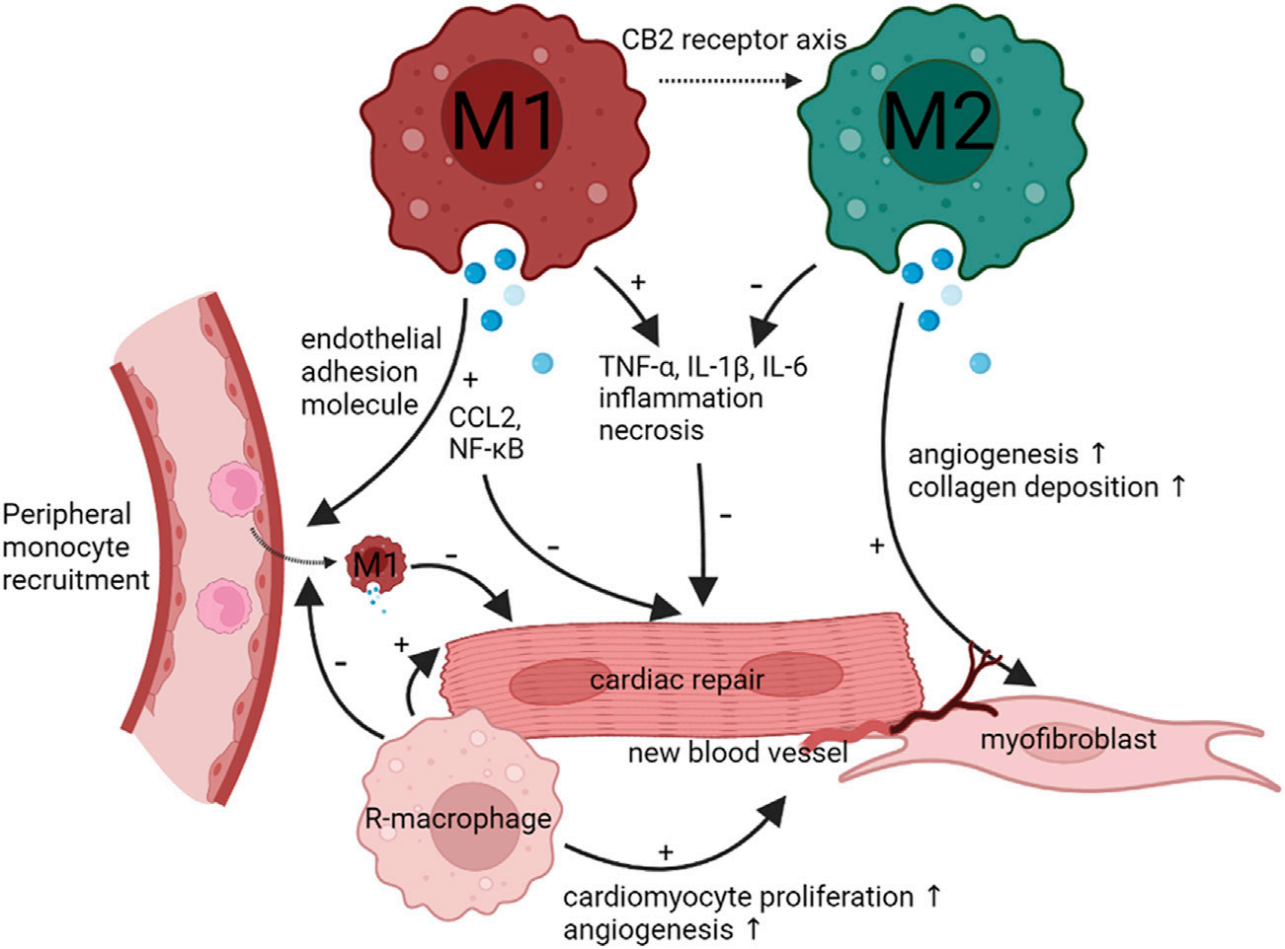
Functions and phenotype of macrophages during infection and tissue repair. When cardiomyocytes undergo injury, the heart recruit peripheral monocyte to differentiate M1 macrophages. M1 macrophages will activate CCL2 and NF-κB and promote TNF-α, IL-1β, and IL-6 to inhibit cardiac repair and aggravate cardiomyocytes necrosis. As time goes on, M1 macrophages can transform M2 macrophages through CB2 receptor axis. M2 macrophages can inhibit TNF-α, IL-1β, and IL-6 to promote cardiac repair. Meanwhile, M2 macrophages can promote angiogenesis and fibroblast formation. Cardiac resident-macrophages have the same effect as M2 macrophages.

The phenotypic switch of resident-macrophages and monocyte-derived macrophages plays a key role in the development and progression in both ischemic and non-ischemic cardiomyopathy (Figure 2). M1-like macrophages exacerbate the inflammation to lead to HF, M2-like macrophages suppress the inflammation. Notably, M2-like macrophages slowly increased. In addition, M1-like and M2-like macrophages rely on the glycolytic and oxidative phosphorylation. Both types of macrophages interact with cardiac other cells such as fibroblasts cells to control disease progression. Thus, regulation of phenotypic transformation of macrophages could be a potential therapeutic target for cardiomyopathy.

Duerr et al. demonstrated that the endocannabinoid CB2 receptor axis protects the ischemic heart by modulating macrophage polarization (M1 to M2), reducing the inflammation and adverse cardiac remodeling after myocardial ischemia-reperfusion (Duerr et al., 2014). Qi Chen et al. also demonstrated that the SR-A1-c-Myc axis may be a promising target for the treatment of DIC by enhancing cardiac repair macrophage proliferation (Zhang et al., 2020). Macrophage migration inhibitory factor is a crucial cardioprotective factor against DIC by promoting autophagolysosome formation. GHSR deficiency aggravates inflammasome in macrophages. It also aggravated ISO-induced myocardial fibrosis and the degree of heart failure, suggesting that GHSR is a potential target for intervention in myocardial fibrosis (Wang et al., 2020). Therefore, controlling the transformation of macrophage phenotype can treat the myocardial injury.

## Conclusion

Significant advances have been made in immune system concerning cardiovascular disease in recent years, especially in maintaining cardiac function. The role of macrophages, as a key immune cell, in cardiomyopathy has been extensively studied. Scientists have found that macrophages play a crucial role in myocardial inflammation and fibrosis, and thus may become a new therapeutic target for cardiomyopathy. However, both the recruitment of peripheral macrophages and the response of cardiac resident macrophages depend on the source of the myocardial damage signal. Immune cells activate fibroblasts to maintain impaired heart function. However, sustained cardiac injury continuously promotes extracellular matrix production, leading to decreased cardiac function and heart failure. Although phenotypic heterogeneity and plasticity of macrophages have been reported in cardiac disease for a decade, the role of M2 macrophages in cardiomyopathy is not fully understood. Therefore, a better understanding of the phenotype, function of macrophages in the cardiomyopathy damage and repair may enhance the development of new therapeutic approaches.
